# Comparison of the 3D-printed operation guide template technique and the free-hand technique for S2-alar-iliac screw placement

**DOI:** 10.1186/s12893-020-00930-5

**Published:** 2020-10-29

**Authors:** Yonghui Zhao, Yulong Ma, Jinlong Liang, Haotian Luo, Xingbo Cai, Yongqing Xu, Sheng Lu

**Affiliations:** 1 Department of Orthopedics, The First People’s Hospital of Yunnan Province, The Affiliated Hospital of Kunming University of Science and Technology, Kunming, 650032 China; 2 Department of Orthopedics, 920 Hospital of the Joint Logistic Support Force, Kunming, 650032 China

**Keywords:** 3D-printed technique, Operation guide template, Free-hand technique, S2AI screws

## Abstract

**Background:**

To compare the safety and accuracy of the 3D-printed operation guide template technique and the free-hand technique in the placement of the S2-alar-iliac (S2AI) screw.

**Methods:**

We conducted a retrospective analysis of 47 patients undergoing S2AI screw placement in our hospital, divided into the 3D-printed operation guide template group and the free-hand screw placement group. The duration of single S2AI screw placement was documented in all patients. A postoperative CT scan was performed to assess screw placement effectiveness according to the distance from the screw tip to the breach of the cortical bone wall.

**Results:**

In total, 42 screws were placed in the guide template group, with an average screw placement duration of 151.6 ± 44.8 s. Screw placement grading was as follows: 40 screws in grade 0, two in grade 1, and none in grades 2 and 3. This grading resulted in excellent and good rates of 95.2% and 100%, respectively. In total, 52 screws were placed in the free-hand group, with an average screw placement duration of 138.3 ± 45.9 s. Screw placement grading was as follows: 42 screws in grade 0, seven in grade 1, three in grade 2, and none in grade 3. This grading resulted in excellent, good and acceptable rates 80.8%, 94.2% and 100%, respectively. Screw placement duration did not significantly differ between the groups (p > 0.05). The excellent rate of screw placement was greater in the guide template group than in the free-hand group (p < 0.05), but the good and acceptable rates did not significantly differ between the two groups (p > 0.05).

**Conclusion:**

Both techniques can be applied to S2AI screw placement. The 3D-printed guide technique is superior to the free-hand technique in terms of safety and accuracy.

## Introduction

Pelvic internal fixation is the key to treating adult spine deformity, severe pelvic inclination, and severe lumbar spondylolisthesis [1]. Due to stress concentrations in the lumbosacral region, unstable distal fixation can lead to postoperative complications such as fixation failure and pseudoarticular formation, especially in patients with osteoporosis [2–4]. The pelvic fixation technique is undergoing continuous improvement. In response to the poor stability of the traditional S1 and/or S2 screw fixation, the Galveston technique improved the strength of the fixation, but it was gradually replaced by iliac screw fixation due to its difficulty of operation [5, 6]. Iliac screw fixation, through the connection module, overcomes the difficulty of fixation in the Galveston technique and further improves the strength of the pelvis fixation [7, 8]. However, the extensive tissue stripping and protrusion of the internal device over the skin not only hinders iliac wing bone grafting [9] but also increases the risk of intraoperative bleeding and infection [10, 11]. To solve the above problems, Sponseller et al. proposed the S2-alar-iliac screw (S2AI) technique and achieved satisfactory results in adult and pediatric orthopedic surgeries [11, 12]. The advantages of this technique include less stripping of the soft tissues, a deeper screw position under the skin, no need for a connecting module during fixation, no hindrance to harvesting the ala of the ilium for grafting, achievement of biomechanical stability similar to that of ilial screw placement, and less intraoperative blood loss, postoperative infection, and pain [13, 14]. In the past decade, this procedure has rapidly evolved and become a focus of research.

Due to the complexity of the pelvic structure, deviations in the position of the screw may cause irreparable harm. Amir et al. [15] conducted an anatomical study on the nerves and blood vessels surrounding S2AI screws in cadaver specimens and found that screw breach of the cortical bone may damage the superior gluteal artery, sciatic nerve, obturator nerve, internal iliac artery and vein, and lumbosacral plexus. Therefore, the safe and accurate placement of S2AI screws is essential.

The approaches of S2AI screw placement have mainly included the free-hand technique, intraoperative navigation, and robot-assisted screw placement [16–19]. The robot navigation system is expensive and involves a long learning curve, so its clinical application has been limited. This study focused on the application of the 3D-printed operation guide template technique and the free-hand technique guided by anatomical signs during operation in S2AI screw placement, comparing their safety and accuracy to provide a reference for clinical application.

## Methods

### Patient selection

We retrospectively analyzed the data of 47 patients treated with S2AI screws in our hospital from March 2015 to December 2018. Before surgery, X-ray and computed tomography (CT) scans were performed to exclude deformity and cancer of the sacrum and pelvis. There were 15 men and 32 women with an average age of 57.8 ± 8.4 years (range: 38–74 years). According to the screw placement technique, the patients were divided into the 3D-printed operation guide template group and the free-hand screw placement group.

The time it took to insert each screw was recorded. All patients underwent CT examination after surgery, and the axial images were used to observe the results of screw placement. The evaluation of screw placement was based on the grading system proposed by Oh et al. [20]. This grading system includes four grades. grade 0: the screw is completely within the cortical bone, without breach of the cortical bone wall; grade 1 (mild): the breach distance is less than 3 mm; grade 2 (moderate): the breach distance is 3–6 mm; grade 3 (severe): the breach distance is greater than 6 mm. The breach distance is defined as the shortest distance from the most distal end of the screw to the adjacent cortical bone. In addition, we defined grade 0/total implanted screws as the excellent rate, (grade 0 + grade 1)/total implanted screws as the good rate, (grade 0 + grade 1 + grade 2)/total implanted screws as the acceptable rate, and grade 3/total implanted screws as the unacceptable rate.

The same surgical team performed all the surgeries in this study. This study was approved by the Ethics Committee of the 920 Hospital of Chinese People's Liberation Army Joint Logistics Support Force. All patients were informed and signed a surgical consent before surgery.

### Preparation of the 3D-printed operation guide template

**Fig. 1:**
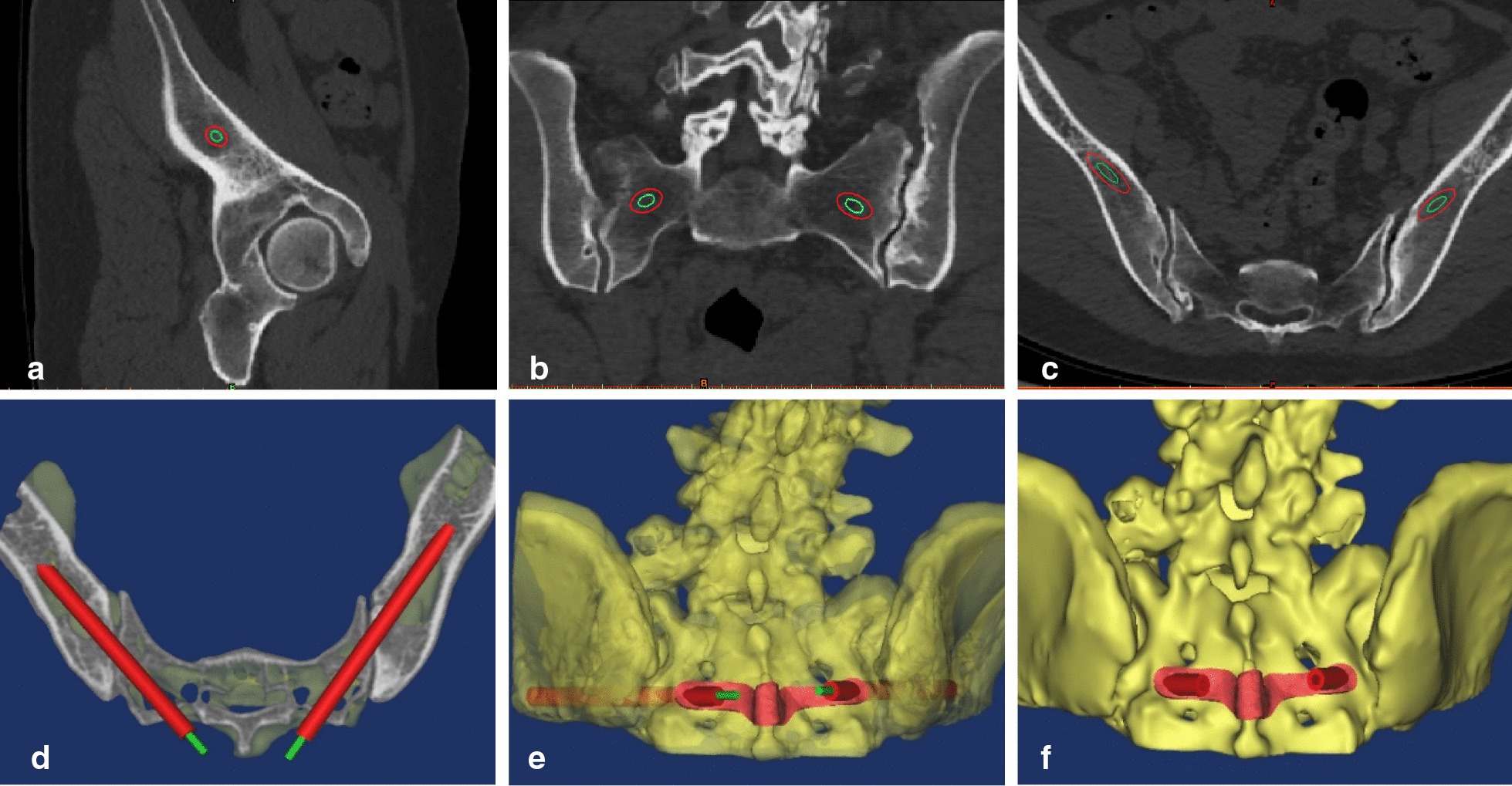
Design of the operation guide template for screw placement. Adjusting the position of the screw trajectory in the sagittal view (**a**), coronal view (**b**), and axial view (**c**). The widest and longest medullary cavity of the ilium in the axial view was selected to design a virtual screw trajectory that ran through the center of the ilium (**d**). Reverse engineering technology was used to extract the shell and construct the attachment surface of the guide plate, and the screw trajectory was accurately aligned with the attachment surface (**e**). Generating a virtual operation guide template in STL format (F)

Before surgery, CT scan of the lumbosacral spine was performed routinely to obtain the raw data in DICOM format, which were imported into Mimics 19.0 software (Materialise, Belgium) to reconstruct three dimensions model of the spine and pelvis. The MedCAD module was used to design the screw trajectory of the template. The screw trajectory was adjusted in the sagittal, coronal, and axial views (Fig. 1a–c). The widest and longest medullary cavity of the ilium in the axial view was selected to design a virtual screw trajectory that ran through the center of the ilium (Fig. 1d). Then, the path of the navigation tube was adjusted properly to avoid damage to the posterior sacral foramina and the sacral canal, if needed. The region bordered by the S1-S2 junction, the median sacral crest, and the posterior sacral foramina was selected for attaching the guide template. Reverse engineering technology was used to extract the shell and construct the attachment surface of the guide plate, and the screw trajectory part was accurately aligned with the attachment surface (Fig. 1e). Then, a virtual operation guide template in STL format was generated (Fig. 1f). Finally, a 3D printer (Shaanxi Hengtong Intelligent Machinery Co., Ltd.) and photosensitive resin 14,120 (DSM Somos, USA) were used to print a physical operation guide template. The residual supportive materials were removed, and then the template was processed using the photocuring technique to enhance its physical properties. Before the operation, the operation guide template was sterilized by the low-temperature-plasma sterilization technique.

### Surgical methods

The posterior midline approach was used for the operation, and the skin and subcutaneous tissue were incised to expose the operation area until reaching the distal second sacral vertebra. The pedicle screws were placed routinely in the upper instrumented vertebra and the first sacral vertebra. Template-guided or free-hand S2AI screw placement was done.

In template-guided screw placement, the soft tissue in the template-attached area was fully stripped, and then the template was securely attached to the bone surface between the median sacral crest and middle sacral crest of the second sacrum. While an assistant held the template in place, the surgeon drilled a hole through the sacroiliac joint along the axis of the screw trajectory using a 2.5-mm drill bit (inner diameter 2.7 mm). Next, a spherical probe was used to check the integrity of the screw trajectory, and a threading tap was used for tapping. According to the size of the screw trajectory measured before surgery, appropriate screws were selected for screw placement, and the screw placement duration was documented.

In the free-hand screw placement, the point 1 mm lateral to the site 2–4 mm below the lower edge of the S1 posterior sacral foramina was selected as the entry point. A grinding drill was used to grind off part of the cortical bone around the entry point, and a pedicle drill was used to make the first approach. While making the approach, the anatomical landmarks were used to determine the tilt angles to the head and lateral side. In the vertical view of direct vision of the surgeon, the tilt angle toward the head for the screw placement was determined according to the projection of the reverse extension line of the screw trajectory in the coronal plane of the human body. That is, the pedicle drill was projected to the site from the lower edge of the superior articular process of the first sacrum to the first posterior sacral foramina on the contralateral side of the insertion point. The orthopedic drill was attached tightly to the upper edge of the superior spine ligament to determine the lateral-tilt angle. The pedicle drill advanced to the sacroiliac joint, and then an orthopedic hammer was used to strike the drill to pass through the joint. A spherical probe was used to explore the four walls and the depth of the bone trajectory. That the four walls were bony walls and the depth was 70–90 mm indicated the achievement of correct screw trajectory. Finally, tapping was performed along the screw trajectory, and then the appropriate S2AI screws were placed. The duration of screw placement was recorded. Intraoperative fluoroscopy of the pelvis (teardrop view or axial view of screw) was performed to finally confirm the position of the screws.

At the completion of the screw placement, spinal canal decompression, tuberculosis lesion removal, and scoliosis correction may be performed according to the patients' condition. We installed a connecting rod to complete the orthopedic fixation. The surgical wound was washed, one or two drainage tubes were routinely placed, and the instruments and gauzes were counted. The operative incision was closed in layers, and the procedure ended.

### Statistical analysis

SPSS 21.0 software was used for data analysis. The Kolmogorov–Smirnov test was used to determine whether the quantitative data were normally distributed. The data with a normal distribution are expressed as $\overline{X} + S$. The independent-sample t test was used to compare the quantitative data between the two groups. The χ^2^ test was used to compare the qualitative data between the two groups. Two tailed α = 0.05 was set as the test level.

## Results

### Comparison of screw results

In the guide template group, 21 sets of individual operation guide templates were designed. All patients underwent S2AI screw placement with assistance of the operation guide template. During all surgeries, the operation guide template fit the bone surface securely, and the operation went smoothly. The duration of single screw placement was 108–340 s, with average of 151.6 ± 44.8 s. Postoperative CT scans were performed to score the screw placement according to the screw grading system and showed 40 screw placements of grade 0, two of grade 1, none of grade 2, and none of grade 3. The excellent rate was 95.2%, and the good rate was 100%. Two of the screws passed through the sacroiliac joint and breached the medial cortex of the ilium.

In the free-hand group, a total of 52 S2AI screws were designed for 26 patients. By referencing anatomical landmarks, we performed free-hand screw placement during each surgery. The duration for single screw placement was 75–243 s, with average of 138.3 ± 44.8 s. Postoperative CT scans were performed to score the screw placement according to the screw grading system and showed 42 screw placements of grade 0, seven of grade 1, three of grade 2, and none of grade 3. Breaching of the screw trajectory was observed in 10 screw placements. Eight of them were located at the medial iliac edge of the sacroiliac joint, and two were at the outer iliac edge of the sacroiliac joint. The excellent rate of screw placement was 80.8%, the good rate was 94.2%, and the acceptable rate was 100%.

The diameter and length of the S2AI screw were 7.0–8.0 mm and 70–90 mm, respectively. Screw breaches from the trajectory were seen in some cases, but there were no related adverse events. There was no significant difference in the screw placement duration between the guide template group and the free-hand group (p > 0.05). The excellent rate of screw placement in the guide template group was greater than that in the free-hand group (p < 0.05), but the good rate and the acceptable rate of screw placement were not statistically significant between the two groups (p > 0.05), as shown in Table 1.

**Table 1:** Comparison of general data and screw placement results between the guide template group and the free-hand group

Group	Number of cases	Sex		Age (years)	Number of screws	Screw placement duration	Screw placement results (%)			
		Male	Female				Excellent rate	Good rate	Acceptable rate	Unacceptable rate
Guide template group	21	6	15	56.9 ± 8.4	42	151.6 ± 44.8	95.2 (40/42)	100 (42/42)	100 (42/42)	0 (0/42)
Free-hand group	26	9	17	58.6 ± 8.1	52	138.3 ± 45.9	80.8 (42/52)	94.2 (49/52)	100 (52/52)	0 (0/52)
Statistic value	–	0.195		− 0.706	–	1.411	4.368	–	–	–
P value	–	0.659		0.484	–	0.162	0.037	0.251	–	–

### Typical case

**Fig. 2:**
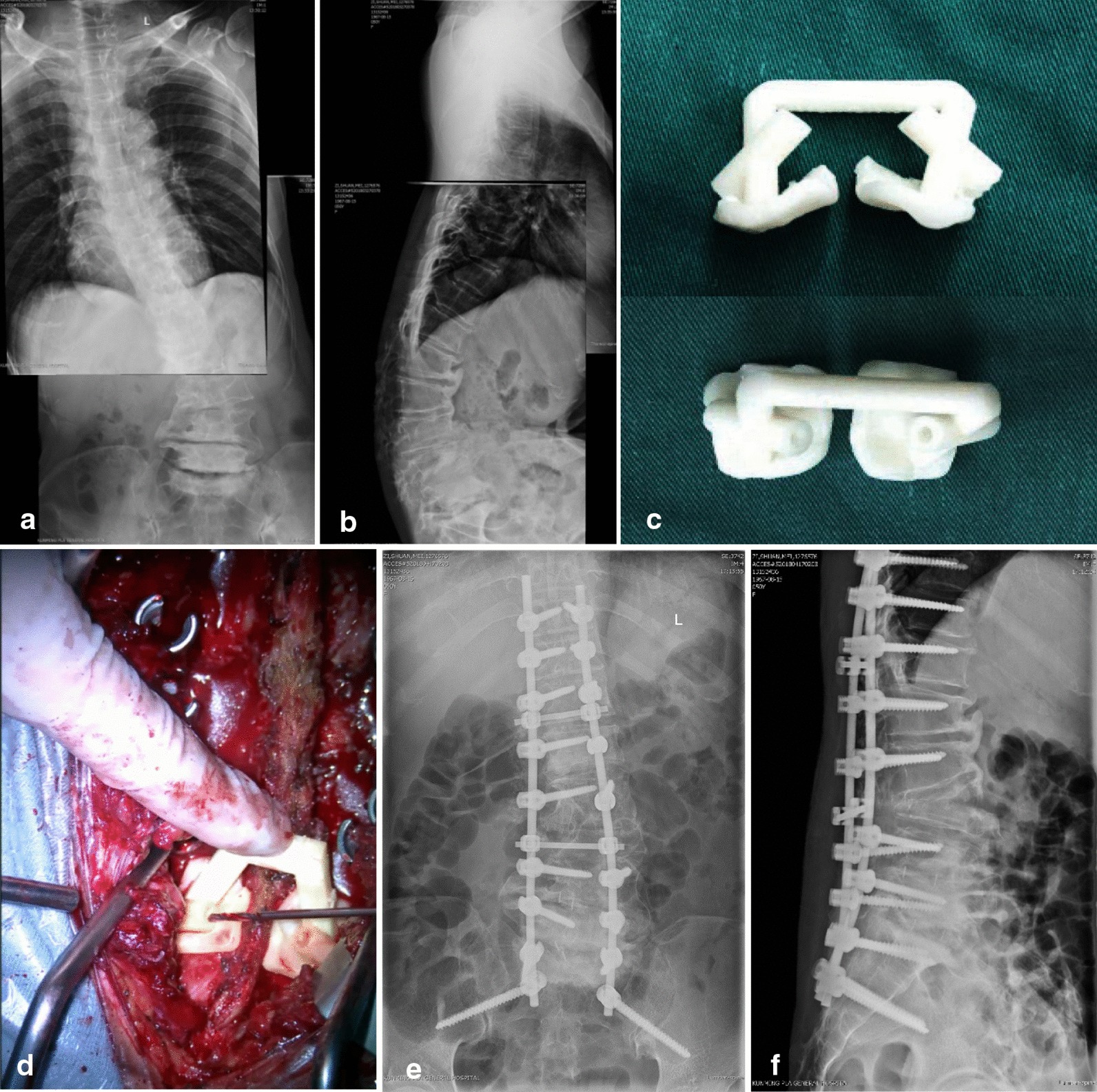
A 53-year-old woman with adult degenerative scoliosis. Anteroposterior-view radiograph (**a**) and lateral-view radiograph (**b**) before surgery. Low-temperature-plasma sterilization of the S2AI operation guide template before surgery (**c**). During the operation, the operation guide template was attached to the bone surface and secured for drilling, tapping, and screw placement along the axis of the screw trajectory (**d**). Postoperative follow-up anteroposterior (**e**) and lateral views (**f**) show that the deformity was well corrected, and the S2AI screw and other screws were in the desired places

The patient was a 53-year-old woman with adult degenerative scoliosis (Fig. 2a, b). Before surgery, the CT data were used to reconstruct the spine and pelvis model and design the personalized S2AI screw operation guide template. The operation guide template model was printed out and disinfected before surgery (Fig. 2c). During the procedure, the T10-S2 segments were exposed, and pedicle screws were used for fixation of the T10-S1 segments. Then, the operation guide template was attached to the bone surface and secured for drilling, tapping, and screw placement along the axis of the screw trajectory (Fig. 2d). According to the condition, L3 vertebral body osteotomy, decompression of the L4-S1 vertebral plate, discectomy, intervertebral bone graft fusion, or internal fixation was selected. Postoperative follow-up radiography showed satisfactory correction of scoliosis and satisfactory positioning of the S2AI screws (Fig. 2e, f). During the 24-month follow-up, the shape of the corrected spine was maintained well, and no other complications were reported.

**Fig. 3:**
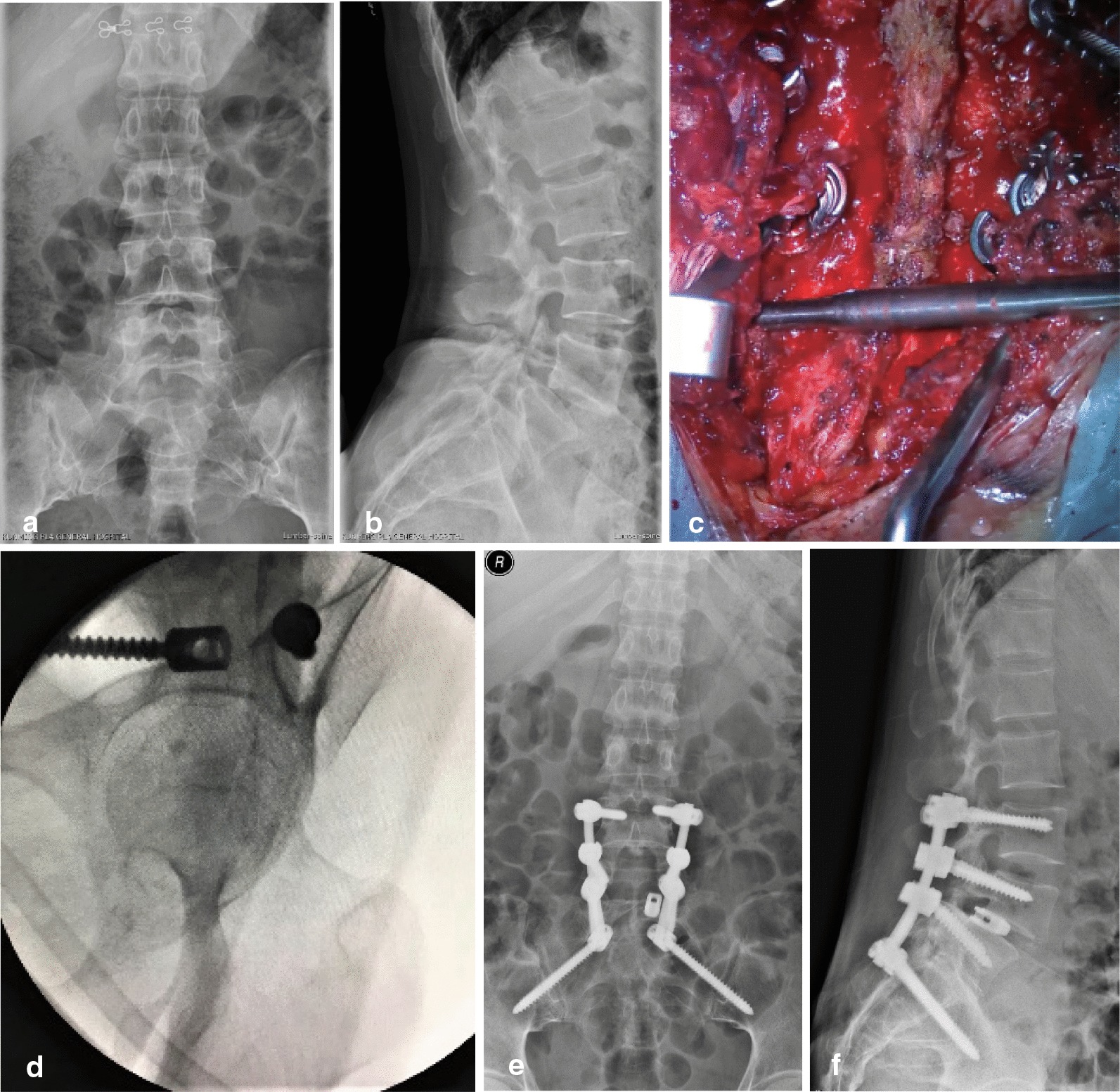
A 38-year-old women with lumbar spondylolisthesis (L5) and unstable spine. Anteroposterior-view radiograph (**a**) and lateral-view radiograph (**b**) before surgery. After selecting the screw entry point, a pedicle drill was projected to the site from the lower edge of the first superior articular process of the first sacrum to the first posterior sacral foramina on the contralateral side of the entry point, and the pedicle drill was close to the upper edge of the supraspinous ligament (**c**). After completion of screw placement, the radiographic teardrop view (axial view of the screw) shows the position of the screw was satisfactory (**d**). Postoperative follow-up anteroposterior (**e**) and lateral views (**f**) show that the slipped vertebrae were reduced, and the S2AI screw and other screws were in the desired places

A 38-year-old man was diagnosed with spondylolisthesis (L5) and spinal instability (Fig. 3a, b). The L4-S2 segments were exposed, and the T4-S1 segments were fixated with pedicle screws. For the S2AI screw placement, the point lateral to the site 2–4 mm below the lower edge of the first posterior sacral foramina was selected as the entry point. Under a vertical view, a pedicle drill was projected to the site from the lower edge of the superior articular process of the first sacrum to the first posterior sacral foramina on the contralateral side of the insertion point, and the pedicle drill was attached to the upper edge of the supraspinous ligament (Fig. 3c). Drilling and tapping were performed while exploring the screw trajectory, and then the appropriate S2AI screws were placed. According to patient’s condition, L4-5 laminectomy, discectomy, slipped vertebra reduction, interbody fusion, or internal fixation was performed when appropriate. At completion of screw placement, intraoperative fluoroscopy (teardrop view or axial view of screw) was performed to confirm the positions of the screws (Fig. 3d). Postoperative imaging examination showed satisfactory reduction in the slipped vertebral body and good positioning of the S2AI screws (Fig. 3e, f). During the 24-month follow-up, the internal fixation stayed in place, and no other complications were reported.

## Discussion

The S2AI screw technique has many advantages, such as reduced trauma, fewer complications, and reliable fixation. Therefore, it has been rapidly developed and applied in spine and pelvic fixation surgery. However, important blood vessels, nerves, and other structures are adjacent to the S2AI screw trajectory, which can lead to disastrous consequences in the case of accidental injury. This study compared two screw placement methods for S2AI screws, namely, screw placement with a 3D-printed operation guide template and free-hand screw placement. After the operation, CT scans were performed to evaluate the two methods of screw placement according to whether the S2AI screw penetrated the screw trajectory and the degree of the screw trajectory.

In free-hand screw placement group, the method we chose was different from that in previous studies [16–18]. In terms of the positioning of the screw entry point, Sponseller et al. [21] suggested that it should be at the site 1 mm from the intersection of the lateral and inferior edges of the posterior sacral foramina. Park et al. [22] chose the site lateral to the midpoint of the connecting line between the S1 and S2 posterior sacral foramina and 2 mm away from the lateral sacral spine. Mattei et al. [23] selected the intersection of the midpoint of the connecting line of the S1 and S2 posterior sacral foramina and the lateral sacral spine. Thus, the entry point for S2AI screws varies. The screw entry point should be chosen to facilitate screw placement and installation of the connecting rod. Changing the entry point will change the screw trajectory. Therefore, the scope of the entry point should be appropriately delimited in the procedure. We selected the point 1 mm lateral to the site 2–4 mm below the lower edge of the posterior sacral foramina as the entry point. The anatomical landmarks were used to determine screw trajectory., i.e., under the vertical view with the naked eye, a pedicle drill was projected to the site from the lower edge of the superior articular process of the sacrum to the first posterior sacral foramina on the contralateral side of the entry point, and the pedicle drill was close to the upper edge of the supraspinous ligament. This method weakens the screw placement angle required for screw placement. During screw placement, it is usually necessary to remove part of the supraspinous ligament and interspinous ligament to avoid the screw tail being blocked during screw placement. Intraoperative fluoroscopy of the pelvis (teardrop view or axial view of screw) was performed to confirm that the screw position was ideal, but postoperative CT still showed screw breach of the cortical bone off the screw trajectory. This finding suggests that there is a certain blind spot in intraoperative fluoroscopy, and the intraoperative fluoroscopic findings cannot be used to assess final screw placement effectiveness. Fortunately, no vascular nerve or other injuries occurred in this study, and no adverse events such as screw loosening were reported during the follow-up.

In recent years, the 3D-printed operation guide template technique has been rapidly evolving in spine surgery. The advantages of the 3D-printed operation guide template include high accuracy, no impact of anatomical signs, low cost, and short production cycle [24, 25]. These are the reasons that we chose the operation guide template to assist in screw placement. A total of 42 S2AI screws were placed in the guide template group. The excellent rate of screw placement was 95.2%, and the good rate was 100%. Two screw breaches in the screw trajectory were noted, but they had no severely adverse impact. A total of 42 S2AI screws were placed in the free-hand screw placement group, with an excellent rate of 80.8%, a good rate of 94.2%, and an acceptable rate of 100%. After the completion of screw placement, there was no significant difference in the time of single S2AI screw placement between the two groups. According to the measurement data, the screw placement time in the guide plate group (151.6 ± 44.8 s) was longer than that in the free-hand group (138.3 ± 45.9 s). This difference is related to the need to take time to carefully remove the soft tissue before using the guide plate and fully expose the bone surface attached to the guide plate. There was no significant difference in the good and acceptable rates between the guide plate group and the free-hand group, but the excellent rate of the guide plate group was significantly higher than that of the free-hand group. This result shows that the 3D-printed operation guide template technique is superior to the free-hand technique in terms of screw placement quality.

It should be noted that there are certain errors in the reconstruction of the spine and pelvis model, operation guide template design, 3D printing, and intraoperative application, which may impact the accuracy of screw placement. Therefore, the inner diameter of the guide navigation tube should be slightly larger than that of the drill bit. If a drill bit with a diameter of 2.5 mm is selected, the inner diameter of the navigation tube should be 2.7 mm. Before using the guide plate, it is necessary to fully expose the attachment surface of the guide plate, so that the guide plate can be fixed firmly, and slowly drill along the navigation tube to avoid a change in the screw trajectory caused by the instability of the guide plate or the sliding of the drill bit. Since the screw placement point is fixed after the guide plate is designed, the screw position of the lower lumbar segment and S1 vertebral body should be considered during the operation so that the screw placement point of the S2AI screw is in a straight line to facilitate fixation of the connecting rod. In addition, although the cost of 3D printing of surgical guide plate technology is decreased and the production cycle is short, the additional cost and time will still be increased. We usually need 1–2 h to design an operation guide plate and 3–5 h to print out the guide plate. Typically, it takes 2–3 days for preoperative planning from the design of the guide plate to the printing of the solid model and preoperative disinfection, which may increase the hospitalization time of patients. Thus, this technique is not suitable for emergency surgery.

At present, 3D printing technology has achieved a satisfactory clinical effect in clinical application. Many large hospitals have digital medical centers according to the needs of clinicians to carry out preoperative planning to assist in the completion of various surgical operations. However, many local hospitals lack of relevant professional technology and equipment, which is also a reason for to further popularize the application of 3D printing technology.

## Conclusion

Both the 3D-printed operation guide template technique and the free-hand technique guided by anatomical landmarks have clinical application value in S2AI screw placement. The 3D-printed guide technique is superior to the free-hand technique in terms of safety and accuracy.

## Data Availability

The datasets generated and/or analyzed during the current study are not publicly available because the data are confidential, but the data are available from the corresponding author on reasonable request.

## Abbreviations

S2AI: S2-alar-iliac
CT: Computed tomography
DICOM: Digital imaging and communications in medicine
STL: Stereolithography
